# Sensor Technologies for Intelligent Transportation Systems

**DOI:** 10.3390/s18041212

**Published:** 2018-04-16

**Authors:** Juan Guerrero-Ibáñez, Sherali Zeadally, Juan Contreras-Castillo

**Affiliations:** 1 Faculty of Telematics, University of Colima, 333 University Avenue, Colima C.P. 28040, Mexico; antonio_guerrero@ucol.mx; 2 College of Communication and Information, University of Kentucky, 315 Little Library Building, Lexington, KY 40506-0224, USA; szeadally@uky.edu

**Keywords:** applications, intelligent transportation systems, sensors, vehicle

## Abstract

Modern society faces serious problems with transportation systems, including but not limited to traffic congestion, safety, and pollution. Information communication technologies have gained increasing attention and importance in modern transportation systems. Automotive manufacturers are developing in-vehicle sensors and their applications in different areas including safety, traffic management, and infotainment. Government institutions are implementing roadside infrastructures such as cameras and sensors to collect data about environmental and traffic conditions. By seamlessly integrating vehicles and sensing devices, their sensing and communication capabilities can be leveraged to achieve smart and intelligent transportation systems. We discuss how sensor technology can be integrated with the transportation infrastructure to achieve a sustainable Intelligent Transportation System (ITS) and how safety, traffic control and infotainment applications can benefit from multiple sensors deployed in different elements of an ITS. Finally, we discuss some of the challenges that need to be addressed to enable a fully operational and cooperative ITS environment.

## 1. Introduction

Transportation systems have become a fundamental base for the economic growth of all nations. Nevertheless, many cities around the world are facing an uncontrolled growth in traffic volume, causing serious problems such as delays, traffic jams, higher fuel prices, increase of CO_2_ emissions, accidents, emergencies, and the degradation of quality of life in modern society. According to a report by the Texas Transportation Institute, in the United States, commuters spend approximately 42 h a year stuck in traffic, drivers waste more than 3 billion gallons of fuel per year, having a total nationwide price tag of $160 billion, equivalent to $960 per commuter [1]. Such problems will worsen in the future because of population growth and the increasing migration to urban areas in many countries around the world as reported by the United Nations Population Fund [2] and the Population Reference Bureau [3]. Hence, there is a strong need to improve the safety and efficiency of transportation.

Advances in Information and Communication Technologies (ICT) in areas such as hardware, software, and communications have created new opportunities for developing a sustainable, intelligent transportation system. The integration of ICT with the transportation infrastructure will enable a better, safer traveling experience and migration to Intelligent Transportation Systems (ITS) which focus on four fundamental principles: sustainability, integration, safety, and responsiveness. These principles will play a fundamental role in achieving the main objectives of the Intelligent Transportation Systems which include access and mobility, environmental sustainability, and economic development [4].

The success of ITS largely depends on the platform used to access, collect, and process accurate data from the environment. Sensing platforms are broadly classified into two categories. The first category is the intra-vehicular sensing platform which collects data about a vehicle’s conditions. The second category, urban sensing platforms, are used to collect information about traffic conditions. Sensor technology is a vital component used for data collection during Vehicle-to-Vehicle (V2V) and Vehicle to Infrastructure (V2I) communications. This data is then provided to transportation management systems for further processing and analysis and subsequent decisions/actions. Smart and intelligent ITS promise to address issues such as high fuel prices, high levels of CO_2_ emissions, high levels of traffic congestions, and improved roads [5,6].

### Research Contributions of This Work

The main contributions of this work are three-fold. First, we describe and discuss how sensor technology can be integrated with the transportation infrastructure to achieve a sustainable intelligent transportation system that addresses issues such as high levels of CO_2_ emissions, high levels of traffic congestions, and improved road safety. We describe where sensors can be placed in the transportation infrastructure, the type of information they collect and how that information is used to improve transportation making it smarter. Second, we discuss how user applications (driving assistance, passenger entertainment, collision alert, among others) and ITS applications (traffic control, road conditions, among others) can benefit from sensor technologies embedded in ITS components. We also present a taxonomy of applications that will directly benefit from all sensor data collected and improve transportation systems by making them more cost-effective, more energy-efficient and operate more efficiently for end-users and transportation systems management organizations. Finally, we discuss some of the challenges that sensor technologies need to address in the future to achieve a fully operational, non-intrusive, cooperative ITS environment.

## 2. Sensor Technology

Over the last decade, sensor technology has become ubiquitous and has attracted a lot of attention. Sensors have been deployed in many areas such as healthcare [7,8], agriculture [9,10], and forest [11,12], vehicle and marine [13,14] monitoring. In transportation, sensor technology supports the design and development of a wide range of applications for traffic control, safety, and entertainment. In recent years, sensors, and actuators such as tire pressure sensor and rear-view visibility systems have become mandatory (due to federal regulation in the United States [15]) in the manufacturing of vehicles and the implementation of intelligent transportation systems, aimed at providing services to increase drivers’ and passengers’ satisfaction, improve road safety and reduce traffic congestion. Other sensors are optionally installed by manufacturers to monitor the performance and status of the vehicle, provide higher efficiency and assistance for drivers. Currently, the average number of sensors in a vehicle is around 60–100, but as vehicles become “smarter”, the number of sensors might reach as many as 200 sensors per vehicle [16].

In [17], the author presents a classification of three categories of sensors based on the place of deployment in the vehicle: powertrain, chassis, and body. Another work classifies sensors in a vehicle based on the type of application the sensor is intended to support, and four categories of sensors are identified: sensors for safety, sensors for diagnostics, sensors for convenience and sensors for environment monitoring [18]. We extend the classification (four categories) proposed in [18] to include two additional categories of sensors, namely sensors for driving monitoring and traffic monitoring, as shown in Table 1.

### 2.1. In-Vehicle Sensors

**Figure 1:**
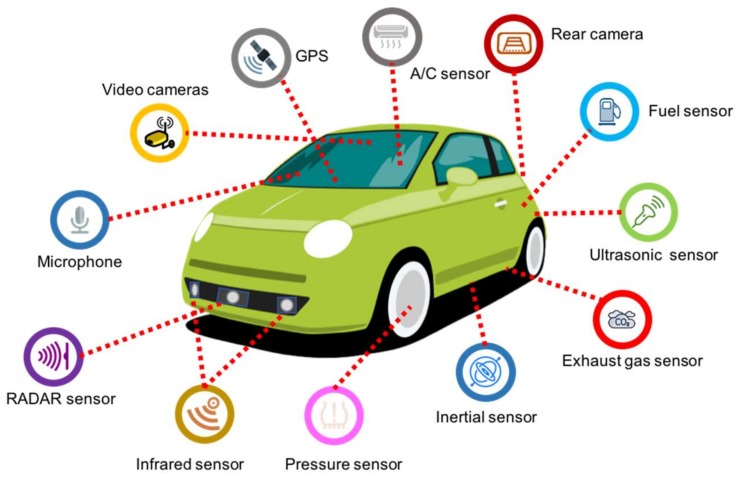
Different types of in-vehicle sensors.

In ITS, identifying the type of sensors to develop applications that contribute to address problems such as: (1) traffic congestion and parking difficulties, (2) longer commuting times, (3) higher levels of CO_2_ emissions, and (4) increase in the number of road accidents, among others is of critical importance for improving a vehicle’s performance as well enhancing the driving experience. Figure 1 depicts some of the most widely used sensors in vehicles today.

#### Applications for In-Vehicle Sensors

Tire-pressure monitoring is an application that is required for the National Highway Traffic Administration of the U.S. to alert drivers using acoustic, light or vibration warning if the tire air pressure is low [19].

Proximity, ultrasonic and electromagnetic sensors are used in parking assistance and reverse warning applications. Proximity sensors can detect when a vehicle gets close to an object. Ultrasonic sensors use a type of sonar to identify how far the vehicle is from an object, alerting the driver when the vehicle gets closer than a set threshold. Electromagnetic sensors alert the driver when an object enters an electromagnetic field created around the front and back bumpers. Proximity sensors have been used to develop a system based on a rectangular capacitive proximity-sensing array for occupant head position quantification to meet the guidelines of the Insurance Institute for Highway Safety (IIHS) [20]. However, these types of sensors are frequently affected by temperature and humidity, reducing their accuracy.

RAdio Detection And Ranging (RADAR) and laser sensors constantly scan the road for frontal, side and rear collisions and allow safety applications to adjust throttle and activate brakes to prevent potential collisions or risk situations by using radio waves to determine the distance between obstacles and the sensor. The application notifies the driver if something close to the vehicle is detected and automatically activates the brakes to avoid a collision.

The gyroscope and accelerometer sensors are used in Inertial Navigation Systems (INS) to determine the vehicle’s parameters such as vehicle position, orientation, and velocity. INS are used in conjunction with Global Positioning Systems (GPS) to improve accuracy.

Radar and speed sensors are used in applications that warn the driver of potential danger if changing lanes or wandering out of the lane is detected. The driver is usually warned through vibration in the seat or steering wheel or acoustically using an alarm.

Cameras are used to: (1) monitor the driver’s body posture, head position and eye activity to detect abnormal conditions such as signs of fatigue or the vehicle behaving erratically (driving out of a straight line on the road or pedestrians crossing suddenly in front of the vehicle) and (2) execute night vision assistance applications to help drivers see farther down the road and detect objects such as animals, people or trees in the path that can cause a potential risky situation or an accident.

LIDAR (LIght Detection And Ranging) has become in a key component for the evolution of autonomous vehicles. LIDAR enables a self-driving car (or any robot) to observe the world with a few special characteristics such as continuous 360-degree visibility and highly accurate depth information. LIDAR sensors continually fire off beams of laser light, and then measure how long it takes for the light to return to the sensor.

Although more sensors are in each vehicle, their integration with other components and the lack of widely accepted standards among different brands is a huge drawback in their adoption. In contrast, current automated systems are limited in their capacities. For example, Volvo’s city safety speed limit is 50 km/h or less to avoid collisions with other vehicles or hitting motorcycles or cyclists. A city safety system is based on a laser unit, so in darkness conditions, the it can only detect a vehicle if its headlights and taillights are on and are clearly visible [21].

### 2.2. In Road Sensors

Strategic investment in transportation infrastructures is vital for a country’s growth and is the central core of a modern economy. Each year, governments worldwide spend a huge amount of money in the transportation sector. In the United States, the yearly investment is around 1.6 percent of the Gross Domestic Product (GDP) [22] and Europe invested around 102 billion euros in 2014 with 52% spent in road infrastructures [23]. Even though the automotive industry has invested a lot of money to increase safety, performance and comfort in vehicles using sensors within the vehicle; traffic data collection using mechanisms located along the roadside has become one of the main challenges for intelligent transportation systems. Sensor deployment within a transportation network provides drivers with new services such as smart parking (e.g., matching drivers with available parking spots) and reduced pricing according to congestion levels on the road. Sensors collect environmental data in real-time which is then processed and analyzed to improve transportation networks and make them resilient.

**Figure 2:**
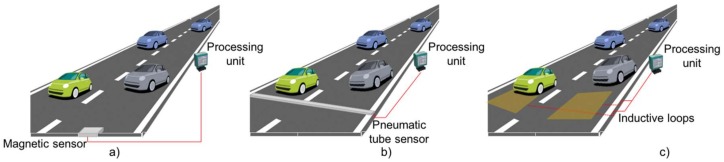
Intrusive sensor groups, (**a**) Embedded magnetometers, (**b**) Pneumatic tube sensors and (**c**) Inductive loops.

Sensors can be classified into two categories based on their location: intrusive and non-intrusive [24]. Intrusive sensors are installed on pavement surfaces. They have high accuracy, but they also have high installation and maintenance costs. Basically, intrusive sensors (as shown in Figure 2) can be classified into three groups: (1) passive magnetic sensors which are installed on roads and are connected either wired or wirelessly to processing units (2) pneumatic tube sensors placed across the road which transmit data to processing units through wired/wireless media, (3) inductive loops that are wire coils buried into roads and send data to processing units. This group of sensors is the most used in traffic control systems [25].

The main advantage of road sensors is their technology maturity. They have been widely implemented and have high accuracy in detecting vehicles. However, the main disadvantages of road sensors are: high installation costs, traffic disruption during installation, maintenance, and repairs. One solution that has been implemented to address the aforementioned drawback is the introduction of wireless battery-powered sensor nodes which replace the intrusive sensors and are installed over the pavement. This technology represents a change in the transportation sensors which are expected to improve the quality, quantity, accuracy, and trustworthiness of the data collected from roads and avenues at a lower cost than current solutions [26,27,28].

**Figure 3:**
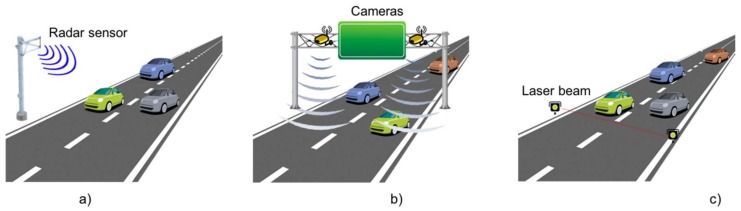
Non-intrusive sensor groups, (**a**) roadside mast-mounted, (**b**) bridge mounted and (**c**) across roadside.

Non-intrusive sensors are installed at different places on the roads (other than over it) as shown in Figure 3 and could detect a vehicle’s transit and other parameters such as vehicle speed, and lane coverage. However, they are expensive and may be affected by environmental conditions. Normally, non-intrusive sensors are used to develop applications that provide information on a selected location, such as queue detection at a traffic light, traffic conditions, weather conditions of the road and the pavement. Some sensors are mounted on a mast and are used to monitor a specific coverage area. Other sensors are mounted on bridges with a monitoring area directly below. Finally, some sensors are placed road side at ground level and use a beam that crosses the road and are mainly used for a single lane and with unidirectional flows because they are very susceptible to interferences from other objects.

Non-intrusive sensors provide many of the intrusive sensors’ functions with fewer difficulties. However, they are highly affected by climate conditions such as: snow, rain, and fog, among others. Accurate traffic data is of utmost importance to make informed decisions to improve traffic conditions. Non-intrusive sensors are more easily spotted by drivers, resulting in different and faster reactions such as: slowing down, and using the correct drive lane, among others after detecting those devices. The challenge is not just the installation of these sensors, but also reducing the drivers’ reactions times based on the collected data and provide them with a more precise view of the context and the reality of the road or avenue.

Currently, several sensors are used on roads. Table 2 shows the two categories (intrusive and non-intrusive) of sensors that are currently used for keeping track of the number of vehicles, vehicle classification, or road conditions [29] as well as some other practical uses.

Pneumatic road tube sensors use one or several tubes placed across traffic lanes allowing for number of vehicles counting and vehicle’s classification. When a vehicle’s tire passes over the tube, the sensor sends a burst of air pressure which produces an electrical signal. The electrical signal is transmitted to the processing unit.

The Inductive Loop Detector (ILD) sensor is one of the most common sensors in traffic management. It is used for collecting traffic flow, vehicle’s occupancy, length, and speed. It consists of a long wire coiled to form a loop which is installed into or under the surface of the road and measures the change in the electrical properties of the circuit when a vehicle passes over the sensor, producing an electrical current that is sent to the processing unit.

Magnetic sensors are used to detect vehicles when a change in the earth’s magnetic field is produced. Magnetic sensors are used to collect flow, occupancy, vehicle length and speed and are suitable for deployment on bridges.

Piezoelectric sensors detect vehicles passing over (at high speed ranges around 112 km/h) a sensor though a change in the sensor’s voltage and can monitor up to four lanes. Piezoelectric systems are commonly formed by piezoelectric sensors and ILDs sensors.

A Video Image Processor (VIP) system includes several video cameras, a computer for processing the images and a sophisticated algorithm-based software for interpreting the images and translating them into traffic data. Video cameras placed at the roadside collect and analyze video images from a traffic scene to determine the changes among successive frames using traffic parameters such as flow volume and occupancy. The main disadvantage of VIP systems is that they are susceptible to reduced performance caused by bad weather conditions.

Radar sensors transmit low-energy microwave radiation that is reflected by all objects within the detection zone. There are different types of radar sensor systems: (1) Doppler systems that use the frequency shift of the return to track the number of vehicles, and determine speed very accurately, (2) frequency-modulated continuous wave radar radiates continuous transmission power such as a simple continuous wave radar and is used to measure flow volume, speed, and presence. In general, radar sensors are very accurate and easy to install. They support multiple detection zones and can operate during the day or night. Their main disadvantage is high susceptibility to electromagnetic interferences.

Infrared sensors detect the energy generated by vehicles, road surfaces or other objects. Basically, sensors convert the reflected energy into electrical signals that are sent to the processing unit. Infrared sensors are divided into two categories: Passive Infrared (PIR) detects vehicles based on emission or reflection of infrared radiation and are used to collect data from flow volume, vehicle presence and occupancy. Active InfraRed (AIR) sensors use Light Emitting Diodes (LED) or laser diodes to measure the reflection time and collect data on flow volume, speed, classification, vehicle presence, and traffic density.

Ultrasonic sensors calculate the distance between two objects based on the elapsed time between a sound wave transmitted at frequencies between 25 and 50 KHz and reflected to the sensor by an object. The received energy is converted into electrical energy which is sent to the processing unit. Ultrasonic sensors are used to collect data about vehicle flow and the vehicle’s speed. The main disadvantage of this kind of sensors is its high sensitivity to environmental effects.

Acoustic array sensors are formed by a set of microphones that are used to detect an increase in sound energy, produced by an approaching vehicle passing through the coverage area of the sensor. Acoustic sensors are replacing magnetic induction loops to calculate traffic volume, occupancy, and average speed of vehicles.

Road surface condition sensors use laser and infrared technologies to read road conditions (temperature and grip) to improve traffic safety and execute road maintenance programs. However, this type of sensor requires periodic maintenance to maintain its performance level.

Radio-Frequency ID (RFID) sensors are used for: (1) automatically identifying running vehicles on roads and collecting their data, (2) for smart parking and for detecting vehicles to allocate space for parking.

Even though many sensors have been installed in roads and streets, the lack of a correct calibration and cluster integration makes the data collected unstable and hinders the development and evolution of ITS as projected and expected from transportation authorities, car makers, road users, and all ITS stakeholders. ITS are expected to use all kinds of integrated sensors to provide situation evaluation systems, and fast decision-making based on the data collected from integrated sensors to improve transportation conditions.

### 2.3. Discussion about Key Sensors

Although sensors are available and widespread, they are a small portion of the various types of equipment used in the automotive industry’s future planning: the self-driving vehicle. The redundancy and integration of sensors will improve the safety and performance of self-driving, automated or autonomous vehicles. Today’s vehicles are already equipped with radar and camera systems, redundant sensors, and software to control them. However, high-resolution and affordable LIDAR systems with ranges up to 300 m and higher are still at the pre-development stages, but it is foreseen that such components will become more advanced in the next few years.

Current camera systems are using CMOS image sensors and machine vision integration with multiple sensors (such as RADAR) for Advanced Driver Assistance Systems (ADAS) and partial autonomous driving. The main disadvantages of camera systems are that environmental conditions can cause problems in the detection of objects in non-illuminated and varying lighting conditions, and computer vision limitations for reliable detection. The challenge of camera systems is generating fast image acquisition and efficient image processing approaches for real-time analysis.

RADAR sensors utilize radio waves to measure the distance and are used with external controllers to modify the throttle to maintain a constant distance from an object. The benefit of RADAR is its low weight and a capability to operate in different conditions, but its main disadvantage is that it has a limited field of vision (small vehicles such as bicycles or motorcycles that are not moving in the center of the lane may not be detected).

LIDAR is a new system in the automotive sector used to measure distances to stationary as well as moving objects. LIDAR employs special procedures to provide three-dimensional images of the objects detected. The main disadvantages of LIDAR are their size, cost, and their limited capabilities in bad weather conditions (snow, fog, rain, and dust particles in the air) because LIDAR uses light spectrum waves. LIDAR is not able to detect a color, or contrast, and yields poor optical recognition. Finally, LIDAR systems today are only very rarely used on a large-scale production. Consequently, the potential of this technology is not yet fully explored because of cost and availability reasons. The challenges for LIDAR technology are to reduce the cost of deployment in all vehicles by reducing their size to enable easy integration in cars and higher aperture angle positions.

Companies such as Tesla are focusing on the development of new vehicles based on systems that contain only cameras and RADAR sensors whereas Google is using LIDAR as the preferred technology in the Waymo project [30].

There is no unique solution for implementing assistance systems in vehicles. The success of the new era of vehicles is based on the integration of multi-sensor systems. Camera systems are being integrated with radar systems (located in the front and back of the vehicle to monitor traffic) to improve the precision of measuring of speed and distance and the detection of the outlines of obstacles and moving objects. Radar does not necessarily give the granularity provided by LIDAR, but Radar and LIDAR are promising technologies that complement each other well.

### 2.4. Interconnection Technologies for ITS

The automotive society [31] has proposed several communication technologies to exchange data among vehicles, transport infrastructure and pedestrians.

#### 2.4.1. Access Technologies for V2V Communications

Modern vehicles are being equipped with many sensors and electronic systems and in-vehicle communication networks. Several protocols and networks are used to communicate the different sensors and devices installed inside the vehicle. Table 3 shows a classification of protocols and communication networks based on different data rate range for safety, traffic control and infotainment applications.

Some of the most predominant access technologies for inter-vehicle and vehicle-to-infrastructure communications are: IEEE Wireless Access in Vehicular Environment (WAVE) standard, which includes the specification of Dedicated Short-Range Communications (DSRC), the IEEE 802.11p for PHY and MAC layers and the IEEE 1609 family for upper layers. These technologies have been studied to evaluate their performance through various PHY and MAC layer optimizations [32,33,34,35,36,37]. DSRC allows sensors, vehicles, and pedestrians to exchange messages over a range of about 300 m. Dynamic Spectrum Access (DSA) is a complementary technology used by DSRC to exchange information over unused spectra [38,39,40,41].

J2735 is another standard developed by the Society of Automotive Engineers (http://standards.sae.org/j2735_200911/), which is used in V2V communications. The standard specifies a set of characteristics (such as messages, data frames, and data elements) used by applications utilizing the DSRC/WAVE-based communications systems.

Even though these technologies have been tested, their poor latency time (around 25 ms) is not fast enough to enable the car to make a collision avoidance action, and, given their transmission frequency, unlicensed spectrum conflicts may arise. Additionally, a potential interference among those technologies (WiFi, WAVE, DSRC, DSA) may occur thereby increasing the possibility of an accident.

#### 2.4.2. Access Technologies for V2I Communications

V2I is based on the wireless exchange of critical safety and operational data between vehicles and the roadside infrastructure to improve the performance of transportation systems. Different access technologies have been proposed for V2I communications.

4G/LTE technology supports high data rate (up to 129 Mbps), low latency, large coverage and high mobility through soft handoff and seamless switching [42,43,44]. Passive magnetic sensors, pneumatic tube sensors and inductive loops can use this technology to disseminate data to vehicles, pedestrians, or traffic control offices.

WiMAX technology provides coverage up to 50 km and supports a data rate of up to 70 Mbps [45], offering broadband access to mobile users for data exchange to enable the implementation of road safety and traffic analysis applications [46,47,48].

In the last few years, research has been focusing on the benefits of using existing and emerging mobile communication standards (such as LTE—X2 interface, and 5G Device-to-Device (D2D) [49,50,51,52,53]) as alternative technologies for delivering automotive applications. The main objective is to use existing telecommunication infrastructures and networks to collect data to provide novel Advanced Driver Assistance Systems solutions.

Recently, SigFox has become a viable solution for exchanging data among IoT devices thereby offering a global Low Power Wide Area (LPWA) network that can be used in ITS for the transmission of data collected from different sensors (pollution level sensors, temperature and humidity sensors, among others) located on roads and avenues [54,55,56].

Wireless technologies can support latency times lower than 5 ms, which could be considered suitable for safety applications [57]. However, the main disadvantage is the high infrastructure cost and the requirement of a subscription or service agreement with carriers for the usage of the network [58].

## 3. Taxonomy of ITS Applications

**Figure 4:**
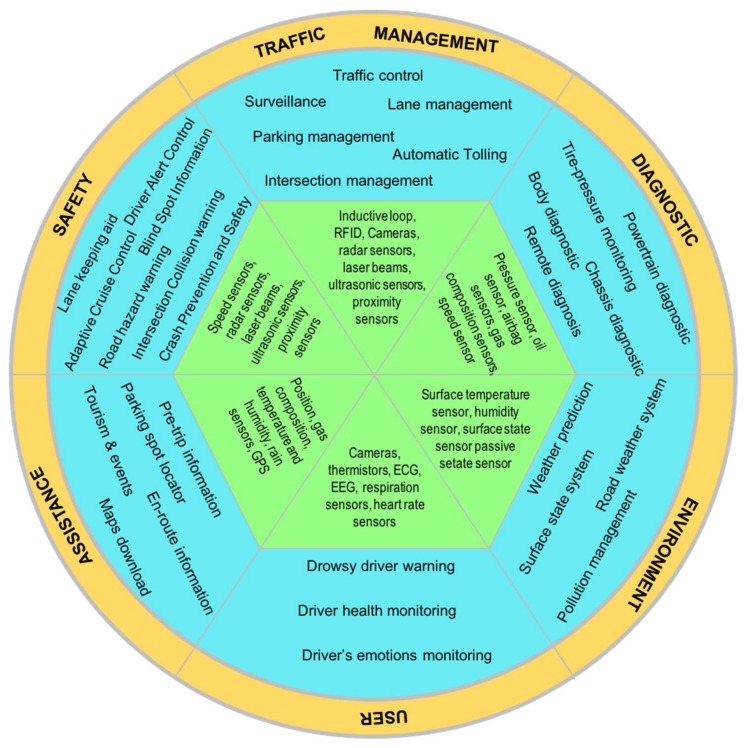
Taxonomy for ITS applications.

Figure 4 presents a taxonomy for ITS applications. The taxonomy defines six categories based on the type of application for ITS.

### 3.1. Safety Category

**Figure 5:**
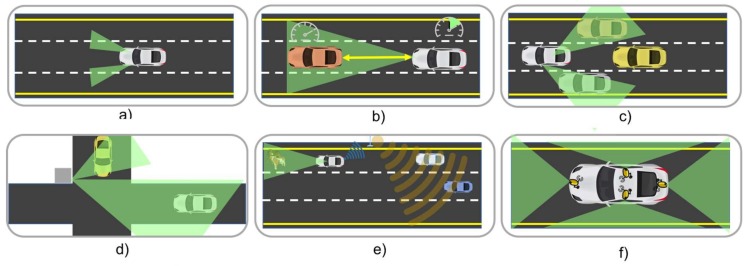
An example of ITS safety applications: (**a**) lane keeping aid, (**b**) adaptive cruise control, (**c**) blind spot information, (**d**) intersection collision warning, (**e**) road hazard warning and (**f**) surround view monitoring.

In the safety category, applications focus on improving the safety of drivers and passengers (as shown in Figure 5) thus reducing the number of accidents, injuries, and fatalities.

An application for lane management focuses on keeping the vehicle safe while driving. Using cameras mounted behind the rear-view mirror the application can monitor road lane markings and detect any drifts outside of a lane [59]. Adaptive cruise control applications use radar, speed, and distance sensors to regulate the speed and maintain a safe distance from the vehicles in front. Sensors are combined with predictive algorithms such as neuro-fuzzy [60,61] or curve radius prediction [62] to determine the best angle for the turn in road curves. Blind spot information is an application that uses radar sensors to alert the driver when changing lanes if a car is detected in the blind spot zone [63,64]. Intersection collision warning applications use position and speed sensors to determine the probability of vehicles colliding and transmits a warning signal when the probability of collision is higher than some established security range. Surround-view monitoring applications use cameras to detect obstacles around the car, making the parking and maneuvering process easier.

To further improve safety applications, it is important to use data fusion from several sources and the use of intelligent processing algorithms to allow not only drivers’ notifications but also to determine reaction times to make fast automated decisions and reduce the potential of road accidents.

### 3.2. Traffic Management Category

**Figure 6:**
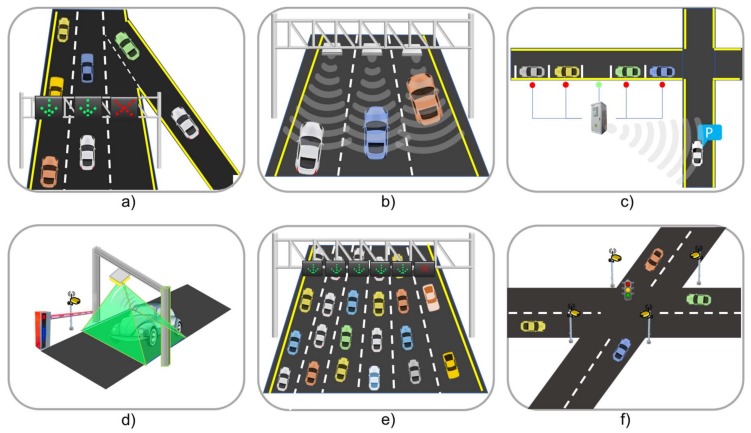
ITS traffic management applications, (**a**) lane management, (**b**) surveillance, (**c**) parking management, (**d**) automatic tolling, (**e**) special event transportation and (**f**) intersection management.

ITS applications in this category improve the traffic flow in roads and urban zones (as shown in Figure 6). Surveillance applications can be divided into two categories: fixed surveillance systems which consist of fixed stations which use cameras and sensors that are installed on the roads to monitor road conditions. The second category, called surveillance-on-the-road, uses sensors and cameras embedded in vehicles to support surveillance [65,66].

Lane management applications focus on managing the available capacity of the roads during special traffic conditions such as emergency evacuations, incidents, or risk weather [67]. This application uses RADAR, cameras, and infrared sensors to detect occupancy, direction, and velocity of vehicles. Special event transportation management systems are a variation of lane management systems and they are used to control and reduce road congestion problems at special places such as stadiums or convention centers. The usage of sensors (such as radar and infrared) and cameras allow a flow direction change in lanes based on traffic demands.

Intersection management applications are cooperative applications that are a viable replacement for traditional traffic light-based methods for intersection control. In this application, road users, infrastructure and traffic control centers work in an integrated way, combining RFID technology, proximity, ultrasonic, radar sensors, cameras, trajectory planning [68], and virtual traffic lights’ modeling, to coordinate traffic safety more efficiently [69].

Parking Management Applications (PMA) use magnetometers, microwaves, inductive loops, infrared or RFID technologies to collect information about parking occupation and inform drivers about parking opportunities or available spaces near their zone [70,71], assisting in the management and distribution of parking spaces thereby reducing traveler frustration and congestion problems associated with searching for parking.

Traffic management applications are becoming increasingly important. However, it is imperative for all those applications to form an integral and collaborative system to enable the deployment of ITS. We need better traffic management with a holistic view of the community and all the stakeholders. For instance, let us assume a city is having a massive event, so the traffic authority decides to establish some rules for a smoother vehicle flow. Lane management application modifies the number of lanes in the same direction, changing from three north-south and three south-north to five north-south and one south-north to optimize arrival to the event, but the core of the problem (smoother vehicle flow) is not solved because most people will look for a place to park, and as the flow increases, the time to find a parking spot becomes more unmanageable. The latter is one of the reasons that calls for a systematic integration of all the applications involved. In this case, lane management and parking management applications could interact to assign an automated parking spot without the need for an on-site decision.

### 3.3. Diagnostic Category

This category focuses on providing diagnostic services that allow the detection of component failures that could lead to a breakdown [72] by using different types of sensors which include: (1) Powertrain sensors to check the status and functioning of the mechanical parts and engine of the vehicle in real time, (2) sensors to monitor fuel level, (3) chemical sensors to check fluid quality, (4) temperature sensors to check the temperature of fluids or gas, (5) composition sensors to monitor engine combustion to reduce pollution, (6) chassis sensors to detect failures in the chassis systems, (7) speed and pressure sensors to monitor the pressure and speed of the wheels that allow the checking of the status of the antilock brake or the traction control system, (8) in-cabin sensors to diagnose the electric and ambience systems. This category can be improved using communication technologies to send information directly to the cloud and to the service and maintenance area of the vehicle. Using a personalized vehicle registry, it is possible to identify and prevent possible car breakdowns by keeping a record and status of each vehicle part.

### 3.4. Environment Category

**Figure 7:**
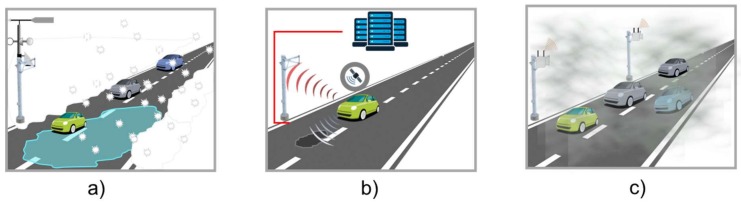
ITS environment monitoring applications, (**a**) road weather condition, (**b**) surface state and (**c**) pollution management.

In the environment category information is collected from sensors deployed in or above the pavement to determine road conditions through the measurement of parameters such as road temperature, road conditions, number of chemical elements and friction or grip of the surface (Figure 7).

Weather prediction applications are based on surveillance, monitoring, and prediction of weather and roadway conditions to implement the appropriate management actions that improve the driving experience and mitigate the impacts of any adverse conditions [73]. Road weather applications are used to facilitate decisions on maintenance strategies and driver advisories. Weather stations and infrared sensors are deployed on roads to determine air temperatures, precipitation, as well as the presence of fog, smoke or other conditions that could increase the risk situations for drivers or affect road maintenance decisions [74].

Road Surface State applications use infrared sensors to measure the infrared radiation emitted by the surface and applying intelligent signal processing to remotely measure road parameters such as: temperature, amount of water, ice, and snow [75]. One variation is the road surface anomalies monitoring application that use sensors such as GPS, laser, infrared, and accelerometers inside vehicle to detect anomalies such as potholes or speed bumps. The collected information is used to create an anomalies map for drivers and help road managers to execute infrastructure maintenance and management operations to ensure safety and comfort to drivers [76].

As mentioned, the individual application work is not successful for the creation of an ITS. Full integration and data exchange supported by cloud computing and intelligent algorithms are crucial for traffic management applications to make decisions considering not only vehicular flow but also environmental conditions and the surroundings to enable a balanced redistribution of traffic and reduce contaminants within a given zone without affecting others.

### 3.5. User Category

**Figure 8:**
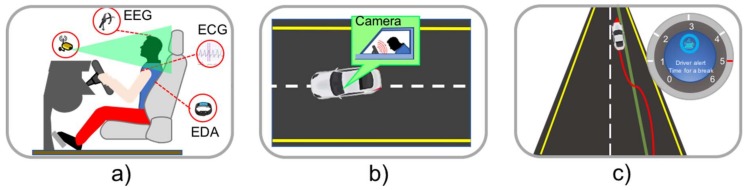
ITS user monitoring applications, (**a**) driver’s health and emotions monitoring, (**b**) drowsy driver warning and (**c**) driver alert control.

In the user category, sensors monitor the drivers’ performance and behavior, which are essential for traffic safety and reducing accidents (Figure 8), using conditions such as: fatigue, alcohol levels and emotional state disorders. The technical report of the American Automobile Association (AAA) identified that drowsy driving caused 21% of deadly traffic accidents and 13% of crashes that required hospitalization [77], drunk driving caused 20% and 69% of fatally-injured drivers in high-income [78] and low-income countries [79] respectively. According to the National Highway Traffic Safety Administration (NHTSA), drivers’ distractions caused 10% of the fatalities in 2014 [80]. Drowsy driving warning applications are used to prevent accidents by monitoring the eyes and head motion using cameras to detect signs of drowsiness. Radar sensors monitor the car’s movements and determine if the vehicle is driven erratically. Other applications can use steering angle sensors to detect an anomaly condition in the driving behavior. The driver is alerted through vibration or audio signals.

Driver alert control applications use front-facing video cameras to track the left and right lane marking to alert the driver if the vehicle drifts outside of those lanes, thus helping reduce the probability of an accident. When the lane is not clearly visible or erased, a camera can be used to monitor the driver by looking for signs of fatigue. The driver is alerted by a sound signal and a flashing message in the control panel of the vehicle [81,82]. Some authors have proposed the use of EEG sensor and Artificial Intelligence (AI) to detect fatigue in the driver by analyzing the EEG brain signals changes through intelligent algorithms to detect abnormal conditions [83,84].

Driver’s health monitoring applications use thermopile, silicon photodiode, optical and infrared sensors with LEDs for measuring vital signs of drivers such as body temperature heart, breathing rates, and blood pressure. Sensors are deployed on steering wheel, seats. When the application detects a problem with the driver health (e.g., a heart attack), an emergency vehicle can be called automatically. Several works have been proposed in the literature that use ECG sensors or wireless bio sensing networks [85,86,87].

Driver’s emotions recognition applications focus on detecting signs of irritation or depression that impair driving. Using Electromyogram (EMG), ECG, respiration, and Electro Dermal Activity (EDA) sensor emotions such as high or low stress, euphoria and disappointment combined with sophisticated algorithms such as Support vector machines (SVMs) and Adaptive Neuro-Fuzzy Interference System (ANFIS), emotions can be detected and classified. In this context, several past works have focused on drivers’ emotion recognition [88,89,90], evaluated emotional states in simulated environments [91,92], and evaluated driver’s stress [93,94] have been published. These applications can be classified into two broad categories: first, car makers need to create non-intrusive sensors for the driver and car occupants’ spots (sensors inside the seat, cameras in strategic spots) to help lessen the burden of the driving task. On the other hand, it is necessary to create intelligent algorithms, based on AI, neural networks, machine learning, computer vision, cloud computing, fog, among others to accurately identify different emotional statuses or abnormal physical conditions in the driver and the passengers to produce trustworthy notifications to the corresponding authorities or designated people.

### 3.6. Assistance Category

Pre-trip information applications collect information about different road conditions, producing several trip options for various driving routes. Parking spot locator applications allow drivers to find available parking places at locations such as streets, garages, or parking lots. Magnetometers, RFID technologies and GPS are used to collect data from different parking spots and can offer drivers a wide variety of opportunities to park their vehicles [95,96,97].

Tourist and events applications are developed to cover the needs of travelers in unknown areas. Drivers are assisted to find the most important places in a city, empty parking slots and routes when drivers travel to major events (sporting games or concerts). Applications use the data collected from sensors (radar sensors, cameras, inductive loops, and weather sensors) deployed near the destination place to calculate travel time and determine alternative routes according to traffic congestion or weather conditions.

On-the-fly routing information uses different sensors (such as cameras, weather sensors, radars, ultrasonic, loops) placed on roads that collect data about traffic conditions to enable users to make informed decisions regarding alternate routes and expected arrival times.

Active prediction applications anticipate the topology of the road to optimize fuel usage and assist drivers by adjusting the speed when the vehicle starts a descent or ascent.

Map download applications help drivers to get valuable information from important places or home stations. Drivers can download travel guidance maps before traveling to a new area to get directions even in location without Internet connection. One disadvantage of this application is its high costs of installation, deployment and maintenance of the data collection, infrastructure (cameras, sensors, among others) and the communication systems to facilitate information transmission to the relevant processing centers. Many crowdsourcing applications such as Waze (http://www.waze.com), Here We Go (http://wego.here.com), and TowIt (http://towit.io) are focusing on mobile devices and the willingness of users to share information to increase the reliability and quality of the data being collected. The processing of this information will help to identify and remove false, incomplete, and redundant information so that users receive accurate information and alerts when they are distributed.

## 4. Case Study Scenario

**Figure 9:**
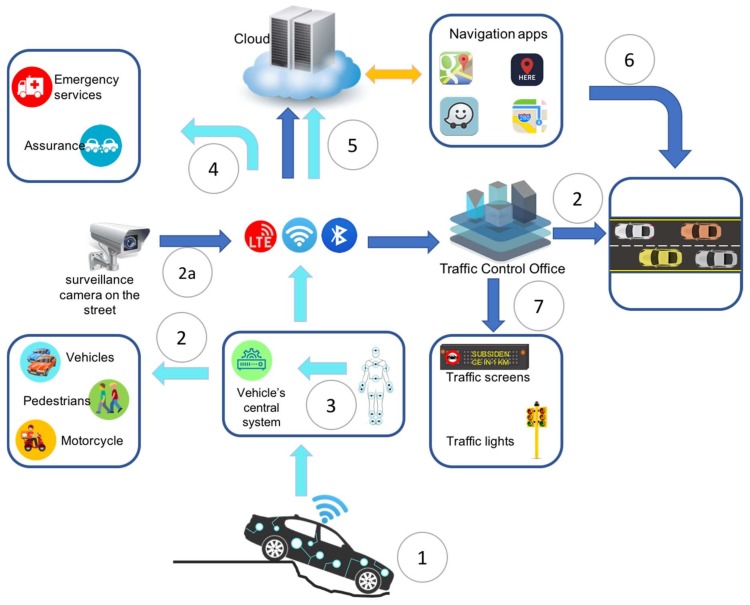
Case study scenario.

In this section, we present a case study that shows how sensing technologies can be integrated with information and communication technologies to improve the transportation systems and provide help and support, for example: (1) when a car is involved in a road accident due to a pothole opening suddenly and the car gets stuck inside (as shown in Figure 9).

(1) The vehicle monitoring system detects a potentially dangerous situation using in-vehicle and outside-vehicle sensors and wearable sensors on passengers (accelerometer, which measures the vehicle’s horizontal position; LIDAR which measures the distance to impact; impact sensors that detect the scale of the impact, ECG which measures the changes in a passenger heart rate, among others), however, the accident is unavoidable and the car gets stuck in a recently formed road cavity. The car immediately starts the included security and safety protocols to perform a preliminary assessment of the situation.

(2) The car’s central system starts a broadcast alert protocol to notify nearby drivers and pedestrians about the accident to take additional safety precautions (in example: reducing speed or taking alternate routes).

(2a) At the same time, using pattern recognition algorithms running in the surveillance cameras, the transport infrastructure detects the situation and activates a set of security measures for this situation such as: intelligent traffic lights can change their light management strategy, prohibiting vehicles from entering the street or blocking road access.

(3) Through wearable sensors on the passenger, the car’s central system receives the information and performs an assessment of passengers’ health status.

(4) After assessing the vehicle’s damages and the passengers’ health status, the central system notifies the relevant parties such as: (i) the vehicle insurer, sending information such as location, insurance policy number and preliminary damage assessment conducted from the information provided by different sensors; and (ii) emergency services, sending the accident notification including, but not limited to, the number of passengers, the passenger location inside the vehicle and vital signals of each passenger, among others.

(5) All the information about the accident, generated by the car’s systems and protocols and the road infrastructure is sent and stored in the cloud and made available for information systems to provide further information and notifications in real time to other drivers.

(6) Location services such as: Google Maps, Apple Maps, Here We Go, and Waze can utilize the information to recalculate new or alternate routes to prevent road congestion or another accident.

(7) The central system sends a notification to the transport infrastructure (traffic lights, warning screens, traffic signals) to continue sending notifications and updates regarding the accident to keep drivers and pedestrians informed about the situation.

## 5. Challenges and Opportunities

Within the scope of intelligent transportation systems applications, we highlight below some challenges that need to be addressed in the future to improve transportation systems, the mobility and safety of drivers and passengers.

Traffic management organizations are aware that intelligent transportation systems face the challenge of how to improve mobility. Hence, they are constantly deploying intelligent sensors within the physical infrastructure and within vehicles and mobile sensing units and systems based on computational vision. However, sensors by themselves cannot solve the mobility challenge. To improve transportation systems, we need the integration of other technologies and devices such as data analytics, automated operation tools, decision-making tools, and social and mobile networks to fulfill the requirements for a complete integration to capture, analyze and share in real time, with the relevant parties, all the information generated by all the different sources.

One of the main challenges faced by intelligent transportation systems is the monitoring of sensing devices’ range located on roads, vehicles, and transportation infrastructures. Currently, sensing systems are faced with a lack of or damaged infrastructure such as: blurry or erased transit lines, inadequate or, in some cases, inexistent traffic signals, fast object detection (pedestrians, cyclists, gravel, tire residues or animals) or risky road conditions (holes in the road, a sudden change in road surface from pavement to another material, and road floods or environmental conditions that could pose a threat to the vehicle’s passengers). However, we must carefully determine where the real constraints (e.g., sensor reachability, the algorithms that process the sensor information) exist. For example, if a vehicle is traveling at a high speed, and the front sensors detect an obstacle (vehicle or object), the algorithms’ information processing time to select the alerting interface or the selected control system is too high, we may not receive the appropriate response in time before the impact. To reduce the likelihood of an accident, different types of sensors, infrared and photogrammetric systems along with efficient algorithms for multisource data fusion could interact and produce better vehicle response times in all driving conditions and improve maps produced in terms of better identifying traffic and road risky conditions [98]. Data fusion techniques have been used extensively in multisensory environments with the aim of fusing and aggregating data from different sensors according to the following criteria:

(a) Input data source relationships: supplementary data, redundant data, or cooperative data [99].
(b) Input/output data type [100].
(c) Architecture: centralized, decentralized or distributed [101,102,103].
(d) The level of data abstraction: raw measurement, signals and characteristics or decisions [104].

The type of data and the different data sources are crucial in addressing many challenges related to transportation systems, such as seamless integration of data from a set of independent data sources that respond in real-time to the needs of actual traffic requirements.

Data fusion techniques must be integrated with AI to allow the vehicle to understand the current state just as a human would and react accordingly, including anticipating different contexts and scenarios [105]. For example, if a driver spots a bouncing ball, the most probable scenario is a boy running after it, so the driver strongly considers stopping the vehicle in immediately. This fusion between sensors and AI could enable the future inclusion of thought inference into the car’s control system to help achieve a faster response from the vehicle to avoid a potential accident situation.

Even though sensors can generate relevant data of transportation systems, the use of additional tools, such as machine learning and data fusion, can generate more data to increase ITS performance, using applications that learn transportation behavior from real-time and historical data [106]. Fusion strategies are used to combine data from different sources to analyze and predict several conditions (such as a driver’s behavior or traffic dynamics) [107]. Machine learning is used to gain knowledge of useful patterns and trends between different traffic data sources based on association rules [108]. The challenge here is the development of efficient learning-driven algorithms that detect and predict traffic patterns that increase the performance of transportation systems. When data samples are collected from different sources (such as camera and sensors) and are transmitted by different media (through wired/wireless links), the challenge is the design and development of algorithms that analyze the data in order to: (i) perform data cleansing to remove abnormal data gathered by sources [109,110]; (ii) remove some redundant features from the original feature space but preserves the interpretability of the remaining features [111,112]; and (iii) compare and fuse the data from different sources [113].

The privacy and security of vehicles highly depend on reducing intrusiveness at two levels. First, to keep the vehicle’s occupants from disclosing any information that could lead to a potential privacy risk, because the sharing of information in a connected environment can enable users to be identified, we should include security and privacy protocols into the communication devices that are supported by the communication networks to protect drivers’ or passengers’ privacy within the vehicular network environment. Additionally, the integration of new devices within vehicles requires them to be optimally placed inside or outside the vehicle so that they do not distract the driver but at the same increase his/her comfort by providing all the relevant information about the different sections of the vehicle. Second, it is also important to consider the optimal interface to alert the driver. One possible solution is to create mechanisms to improve the drivers’ focus on driving; minimize the distractors, including alerts and roadside infrastructure information, possibly by automating some tasks based on human inference through AI.

Creating 360° vision for the driver requires the integration of different devices within the vehicle to increase the detecting range and the precision of the detection by using a multitude of information sources. However, to achieve this objective, the hardware and software costs involved will be high. Additionally, a large amount of information will be generated. The processing power required to calculate the alert could be high. Consequently, we need to develop optimal processing algorithms to select the best interface to alert the driver. We need a careful trade-off between the number of sensing devices and the number of alerting devices while maintaining a competitive price for the vehicle because high costs will impact sales for the vehicle. One approach is to increase the speed at which objects can be detected and improve the level and range of vision of the moving vehicle [114,115,116]. Such improvements will enable a faster and more appropriate response to emergency situations.

## 6. Conclusions

Sensors will play a vital role for ITS in the future. Their usage enables the development of a wide variety of applications for traffic safety, traffic control entertainment and driver assistance. Sensors provide the mechanism to data acquisition related to the vehicular context (such as road conditions, traffic conditions, vehicle conditions) that can be integrated with the current transportation systems to mitigate some of the problems that past and current transportation systems have been facing. The use of analytical and statistical techniques demonstrates the real potential of integrating sensors with ITS. This integration is a promising research area that will broaden the development of a wide range of next-generation smart applications aimed at improving the safety and traffic control of existing and future transportation systems.

## Figures and Tables

**Table 1:** Classification of sensors used in a vehicle (based on [18]).

Category of Sensors	Description	Example
Safety	Form the basis of safety systems and focus on recognizing accident hazards and events almost in real-time.	Micro-mechanical oscillators, speed sensors, cameras, radars and laser beams, inertial sensors, ultrasonic sensors, proximity sensors, night vision sensors, haptic.
Diagnostic	Focus on gathering data for providing real-time information about status and performance of the vehicle for detecting any malfunction of the vehicle.	Position sensor, chemical sensors, temperature sensors, gas composition sensors, pressure sensor, airbag sensor.
Traffic	Monitor the traffic conditions in specific zones, gathering data that improves the traffic management.	Cameras, radars, ultrasonic, proximity.
Assistance	Responsible for gathering data that provide support for comfort and convenience applications.	Gas composition sensor, humidity sensors, temperature sensors, position sensors, torque sensors, image sensors, rain sensors, fogging prevention sensors, distance sensors.
Environment	Monitor the environment conditions, offering drivers and passengers alert and warning services that are used to enhance their trips.	Pressure sensors, temperature sensors, distance sensors, cameras, weather conditions.
User	Focus on gathering data that support the detection of abnormal health conditions and behavior of the driver that can deteriorate the driver’s performance.	Cameras, thermistors, Electrocardiogram (ECG) sensors, Electroencephalogram (EEG). sensors, heart rate sensor.

**Table 2:** Categories of sensors currently used for traffic control.

Category	Sensor Type	Application and Use
Intrusive	Pneumatic road tube.	Used for keeping track of the number of vehicles, vehicle classification and vehicle count.
	Inductive Loop Detector (ILD).	Used for detection vehicle’s movement, presence, count and occupancy. The signals generated are recorded in a device at the roadside.
	Magnetic sensors.	Used for detection of presence of vehicle, identifying stopped and moving vehicles.
	Piezoelectric.	Classification of vehicles, count vehicles and measuring vehicle’s weight and speed.
Non-intrusive	Video cameras.	Detection of vehicles across several lanes and can classify vehicles by their length and report vehicle presence, flow rate, occupancy, and speed for each class.
	Radar sensors.	Vehicular volume and speed measurement, detection of direction of motion of vehicle and used by applications for managing traffic lights.
	Infrared.	Application for speed measurement, vehicle length, volume, and lane occupancy.
	Ultrasonic.	Tracking the number of vehicles, vehicle’s presence, and occupancy.
	Acoustic array sensors	Used in the development of applications for measuring vehicle’s passage, presence, and speed.
	Road surface condition sensors	Used to collect information on weather conditions such as the surface temperature, dew point, water film height, the road conditions and grip.
	RFID (Radio-frequency identification)	Used to track vehicles mainly for toll management.

**Table 3:** Classification of communication protocols and communication networks for intra-vehicle communication.

Data Rate	Application Domain	Protocols and Communication Networks
Less than 10 Kb/s	Control data used for driving and passenger monitoring.	Local Interconnect Network (LIN), Time-Triggered Light Weight Protocol (TTP/A).
10–25 Kb/s	General data (temperature, humidity, sound level, among others) not related to diagnostic or critical information.	Controller Area Network-Bus (CAN-B), J1850.
125 Kb/s–1 Mb/s	Transmission of information related to powertrain and chassis.	Controller Area Network-Bus (CAN-B).
Higher than 1 Mb/s	Multimedia and infotainment applications.	Media Oriented System Transport (MOST), Digital Data Bus, Bluetooth, FlexRay, ZigBee, WiFi and Ultra-wideband (UWB).
